# Comparative outcomes of total wrist arthrodesis for salvage of failed total wrist arthroplasty and primary wrist arthrodesis

**DOI:** 10.1177/17531934211057389

**Published:** 2021-11-19

**Authors:** Hero J. A. Zijlker, Ruben K. Fakkert, Annechien Beumer, Cees B. IJsselstein, Mascha Wessels, Marco J. P. F. Ritt

**Affiliations:** 1 Department of Plastic, Reconstructive and Hand Surgery, Amsterdam UMC, Amsterdam, The Netherlands; 2 Department of Orthopaedic Surgery, Amphia Hospital, Breda, The Netherlands; 3 Coronel Institute of Occupational Health, Amsterdam UMC, Amsterdam, The Netherlands; 4 Department of Plastic, Reconstructive and Hand Surgery, Albert Schweitzer Hospital, Dordrecht, The Netherlands; 5 Department of Radiology, Reade, Amsterdam, The Netherlands

**Keywords:** Wrist, arthroplasty, arthrodesis, plate fixation, salvage, revision

## Abstract

A retrospective study compared outcomes of total wrist arthrodesis as a salvage for total wrist arthroplasty versus primary total wrist arthrodesis. Seventy-one wrists were reviewed after a minimum follow-up of 12 months. Thirty-two wrists with failed total wrist arthroplasty were converted to a wrist arthrodesis and 39 wrists received a primary wrist arthrodesis. Seven converted wrist arthrodeses and five primary arthrodeses failed to fuse. Mean patient-rated wrist and hand evaluation scores and work-related questionnaire for upper extremity disorders scores were 43 and 39 for converted total wrist arthrodesis and 38 and 33 for the primary total wrist arthrodesis. Overall, there were 25 complications in 15 patients in the converted wrist arthrodesis group and 21 complications in 16 patients after a primary wrist arthrodesis. The results between the two groups were slightly in favour of patients with a primary wrist arthrodesis. Therefore, we conclude that the timing, primary or conversion, of total wrist arthrodesis could influence patient outcomes.

**Level of evidence:** III

## Introduction

Rheumatoid arthritis and primary or post-traumatic osteoarthritis can cause degeneration of the wrist, which results in pain, limited range of motion and reduced grip strength. Total wrist arthroplasty (TWA) aims to treat pain while preserving motion of the affected wrist. However, especially when compared with total knee and hip implants, the durability of a total wrist implant is still suboptimal. Frequent mechanisms of failure include implant wear and loosening. Failed TWA can be salvaged by revision implant replacement or conversion to a total wrist arthrodesis (Zijlker et al., 2019). The latter comes with technical difficulties, such as severe bone loss after implant removal and poor quality of bone, especially in rheumatoid patients. Therefore, the use of large structural grafts is needed to fill up the bone defect. The purpose of this salvage operation is to achieve wrist stability and minimize pain. Literature on the clinical outcomes of total wrist arthrodesis used as a salvage technique remains scarce with only reports of small series (Adams et al., 2016; Reigstad and Rokkum, 2014; Reigstad et al., 2017). The aim of this retrospective study was to evaluate the radiographic and clinical results of a consecutive series of salvage total wrist arthrodesis for failed TWA, and to compare these results with those of a primary total wrist arthrodesis.

## Methods

### Study design

This study was conducted retrospectively. A database search identified patients who received a total wrist arthrodesis between 2008 and 2019 at the Albert Schweitzer Hospital in Dordrecht and the Amphia Hospital in Breda, both in the Netherlands. Patients were informed about the study and a questionnaire was sent after obtaining informed consent. The following information was extracted from the medical records: reason for the total wrist arthrodesis (as salvage for failed total wrist arthrodesis or as a primary operation); demographics; perioperative findings; radiographs; complications and re-operations. Patients who were lost to follow-up, who could not be reached, with a follow-up less than 12 months, with hemiparesis of the affected arm or with incomplete radiographic follow-up were excluded. The primary outcome measure was the initial rate of bone fusion. Secondary outcome measures were patient-related outcome measures (PROMs), complications and re-operations. The study was approved by the Medical Ethics Committee at the Albert Schweitzer Hospital in Dordrecht, The Netherlands, and Amphia Hospital in Breda, The Netherlands.

### Surgical techniques and postoperative treatment

All operations were performed by two senior authors (AB and CBIJ) with an experience Level IV according to the classification by Tang and Giddins (2016). Preoperatively, all patients received a single dose of prophylactic antibiotics. A tourniquet (250–300 mmHg) was applied around the lower arm.

### Wrist arthrodesis following failed TWA

**Figure 1.**
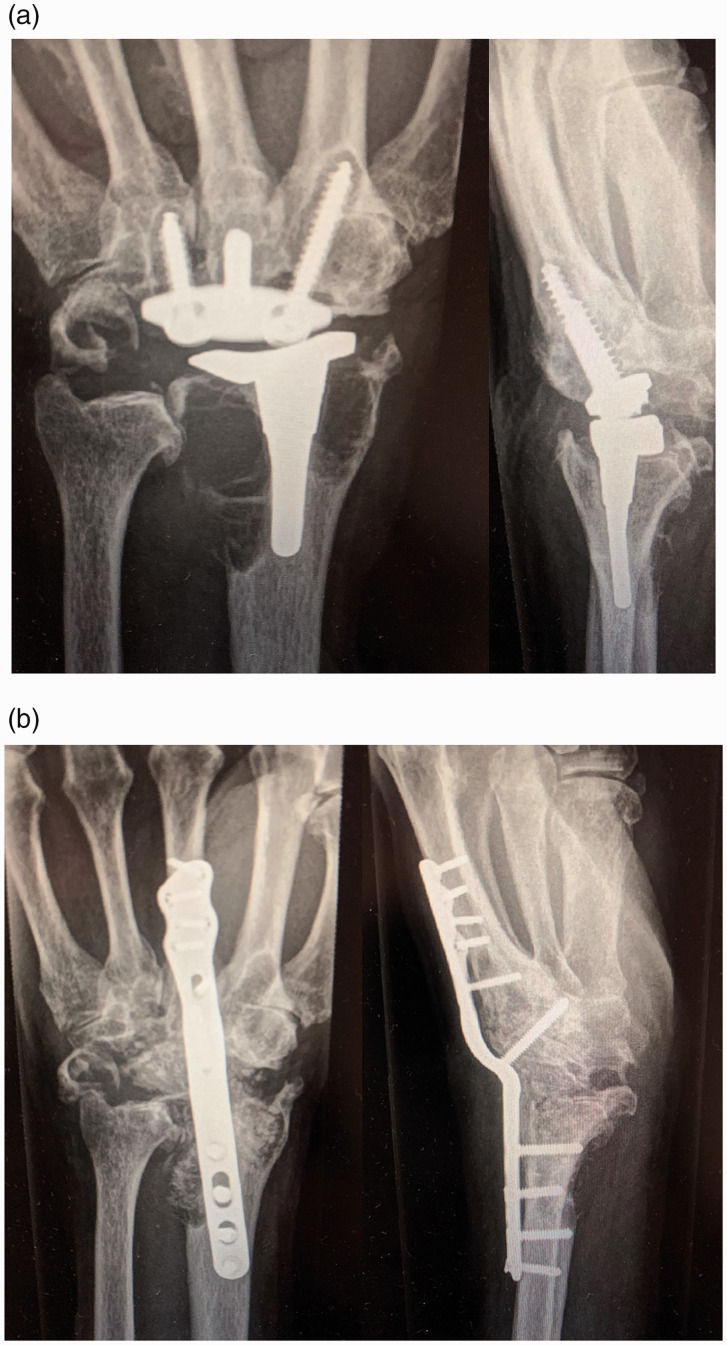
Lateral and anteroposterior radiographs of (a) a failed Universal 2 wrist implant and (b) after revision to total wrist arthrodesis.

In patients with a converted total wrist arthrodesis for failed TWA, the wrist was approached through the previous dorsal skin incision. The third and fourth extensor compartments were opened with a Z-shaped incision and the exposed dorsal wrist capsule was opened longitudinally. The extensor pollicis longus (EPL) tendon was retracted radially and the tendons of the fourth extensor compartment were retracted ulnarly. The posterior interosseous nerve was resected routinely, if not resected previously. The failed TWAs (Biaxial implants,* n* = 8 (BIAX, DePuy, Inc., Warsaw, IN, USA); Universal 2 implants, *n* = 23 (Integra, Plainsboro, NJ, USA) and Remotion implant, *n* = 1 (ReMotion, Small Bone Innovation, Morristown, PA, USA)) were removed with care to preserve bone stock. Thorough synovectomy and debridement were performed. One surgeon (AB) performed a 2-stage wrist arthrodesis for failed TWA. During the first session, the failed implant was removed, followed by debridement and collections of specimens for bacterial cultures and histology, after which a spacer, made of gentamycin-loaded bone-cement, was placed to fill the defect. During the second operation the spacer was removed and cancellous core from a pulverized femoral head allograft (*n* = 7) was used to fill the bony defect. The other surgeon (CBIJ) performed the conversion to a total wrist arthrodesis in one session and used an autologous corticocancellous bone graft from the iliac crest (*n* = 25), which was shaped to engage with the distal radius proximally and the remains of the carpal bone stock distally. Fixation was carried out with a standard dorsal plate (*n* = 29) (DePuy Synthes, Blackpool, UK, *n* = 25; Acumed, Hillsboro, Oregan, USA, *n * = 4) (Figure 1) or an intramedullary Steinmann pin (*n = *3). The dorsal wrist capsule was put over the middle of the plate and extensor retinaculum was closed. The overlying skin was closed over a drain, which was removed the next day.

### Primary wrist arthrodesis

In patients with a primary total wrist arthrodesis, the wrist was approached through a dorsal longitudinal skin incision. The third and fourth extensor compartments were opened with a Z-shaped incision and the exposed dorsal wrist capsule was opened and radially based. The intercarpal ligaments were divided and the articular surfaces of the radiocarpal and intercarpal joints were decorticated. Autologous cancellous bone graft from the iliac crest (*n* = 8), the carpal bones (*n* = 24) or the distal radius or ulna (*n* = 7) was placed around the decorticated surfaces. The dorsal prominences of the distal radius, lunate, capitate and proximal third metacarpal were resected. All wrists were fixed with a titanium dorsal plate (*n* = 39) (DePuy Synthes, Blackpool, UK, *n* = 31; Acumed, Hillsboro, Oregan, USA, *n* = 8).

Postoperatively, for both groups, a cast was worn for a period of 6 to 8 weeks followed by hand therapy. Finally, a custom-made splint was fabricated and used until there was radiographic evidence of bony fusion.

### Radiographic assessment

Standard posteroanterior and lateral radiographs were taken during follow-up to determine bone fusion. Some patients underwent an additional computed tomography scan. Information about bone healing was collected from the radiology report and the clinical file. Bone fusion was defined as partial visible bridging of trabecular bone between the graft and the distal radius and between the lunate and capitate bone in the primary arthrodesis group.

### Clinical evaluation

After verbal agreement, questionnaires were conducted by telephone or sent by email as final follow-up. The PROMs were assessed using the Patient-Rated Wrist and Hand Evaluation (PRWHE; Dutch version) (MacDermid, 1996) and the Work-Related Questionnaire for Upper extremity disorders (WORQ-UP; Dutch version) (Aerts et al., 2017). Additionally, patients were asked how satisfied they were with the result after wrist arthrodesis, using a scale from 0 (not satisfied) to 10 (fully satisfied). Complications and re-operations were recorded from the clinical records. Complications were defined as ‘early’ within 6 months after surgery and as ‘late’ after 6 months.

### Statistics

Descriptive statistics were used to summarize demographics and clinical variables. Frequencies are provided as absolute values and percentages. Gaussian variables are presented as means with standard deviations and non-Gaussian variables are presented as medians with range.

## Results

### Follow-up and demographics

The database search identified 121 patients (124 wrists), 46 (49 wrists) with a converted total wrist arthrodesis and 75 (75 wrists) with a primary wrist arthrodesis. Fifty patients (53 wrists) were excluded: 23 patients could not be reached,12 declined participation, 11 with a follow-up less than 12 months, two had incomplete follow-up radiographs and two had a hemiparesis of the affected arm. Seventy-one patients (71 wrists) were included in the study (36 from the Albert Schweitzer Hospital and 35 from the Amphia Hospital). Thirty-two patients (32 wrists) underwent a converted wrist arthrodesis with a mean follow-up of 68 months (SD 34, range 14–130). The indication for conversion to an arthrodesis included implant loosening in 22 wrists, persistent pain in four, polyethylene wear in two, recurrent subluxation or dislocation in two and infection in two. Thirty-nine patients (39 wrists) underwent a primary wrist arthrodesis with a mean follow-up of 57 months (SD 28, range 14–133). Sample characteristics and the initial diagnosis at time of surgery are listed in Table 1.

**Table 1.** Sample characteristics and initial diagnosis.

		Total wrist arthrodesis for failed TWA (32 wrists)	Primary total wrist arthrodesis (39 wrists)	Total (71 wrists)
Mean age (years) (SD, range)		60 (11, 41–88)	60 (11, 42–83)	60 (11, 41–88)
Male, female		10, 22	24, 15	34, 37
Rheumatoid disease		20	7	27
Non-rheumatoid disease		12	32	44
Smoking	Yes, No	8, 24	10, 29	18, 53
DMARD	Yes, No	19, 13	8, 31	27, 44
ASA class	1	7	9	16
	2	19	28	47
	3	6	2	8
	4	0	0	0

TWA: total wrist arthroplasty; DMARD: disease-modifying anti-rheumatic drugs; ASA: American Society of Anesthesiologists.

### Radiographic evaluation

Based on radiological results, fusion was achieved in 26 of 32 wrists in the converted wrist arthrodesis group and in 34 of 39 wrists (87%) in the primary arthrodesis group. However, non-union became evident after plate removal in an additional patient who had had converted wrist arthrodesis; hence finally union was achieved in 25 of 32 wrists (78%).

### Clinical evaluation and patient reported outcomes

Sixty-nine patients (97%) completed the questionnaires. The mean PRWHE and WORQ-UP scores were 43 (SD 25) and 39 (SD 18), respectively, for patients in the converted arthrodesis group and 38 (SD 29) and 33 (SD 18), respectively, for patients in the primary arthrodesis group. Total absence of pain during activity was reported by seven patients in the converted arthrodesis group and by nine patients in the primary arthrodesis group. The median reported satisfaction score was 7 (range 1 to 10) for the converted arthrodesis group and 8 (range 3 to 10) for the primary arthrodesis group.

### Complications and re-operations

The early and late complications are presented in Table 2. Overall, there were 25 complications in 15 patients (47%) in the converted arthrodesis group; eight were early and 17 were late. These complications resulted in 17 additional operations in 15 patients: five plate removals, one treatment of a deep infection, three carpal tunnel decompressions, one EPL tendon reconstruction, six tenolysis, one distal radioulnar joint (DRUJ) arthroplasty, two stabilizations of the ulnar stump and seven revision wrist arthrodeses because of nonunion (*n* = 6) or pseudoarthrosis after plate removal (*n* = 1). Of the seven wrists that failed to fuse, three had received femoral head allografts (three of seven) and four received iliac crest autograft (four of 25). There were 21 complications in 16 patients (41%) in the primary arthrodesis group; six were early and 15 were late. This resulted in 14 additional operations in 12 patients: eight plate and screw removals, one DRUJ arthroplasty, two EPL tendon reconstruction, two tenolysis and five revision wrist arthrodeses for nonunion. In both groups some procedures were performed during the same operation.

**Table 2.** Early and late complications.

Complications	Total wrist arthrodesis for failed TWA	Primary total wrist arthrodesis
*Early*	*n* = 8	*n* = 6
Complex regional pain syndrome	0	1
Carpal tunnel syndrome	2	0
Hardware failure	0	1
Deep infection	1	0
Tendon irritation/adhesions/rupture	4	3
Donor site seroma	1	0
Ulnar neuropathy	0	1
*Late*	*n* = 17	*n* = 15
Nonunion	7 ^a^	5
Tendon irritation/adhesions/rupture	5	5
Hardware failure	2	1
Carpal tunnel syndrome	1	1
DRUJ problems	2	1
Pain due to screw or plate	0	2

TWA: total wrist arthroplasty; DRUJ: distal radioulnar joint.

a One wrist with pseudoarthrosis after plate removal.

## Discussion

Our data shows a high complication rate in both groups with a slightly higher rate of bone fusion and slightly more favourable PROMs in patients with a primary wrist arthrodesis compared with patients with a converted arthrodesis, although it is uncertain if the differences are statistically significant or clinically relevant. A variety of techniques have been described for conversion of failed total wrist implants to arthrodesis with different rates of bone fusion, namely: (1) a technique proposed by Beer and Turner (1997) using intramedullary Steinmann pins, staples and an iliac crest autograft led to poor fusion rates with union in seven out of 12 wrists (58%); (2) Rizzo et al. (2011) utilized a similar technique with Steinmann pins or a locking plate and a structural graft and achieved fusion in 11 out of 21 wrists (52%); (3) a technique described by Carlsson and Simmons (1998) with Steinmann pins and a femoral head allograft or iliac crest autograft reported fusion in all 12 wrists (100%); (4) Adams et al. (2016) reported fusion in 19 out of 20 wrists (95%) after a median of 4 months using a dorsal plate and a bone-grafting technique described by Carlson and Simmons (1998); (5) Reigstad et al. (2017) demonstrated successful conversion to fusion in all 11 wrists (100%) using a dorsal plate or a customized peg and an iliac crest autograft. A possible explanation for these different rates of fusion might be the variety of techniques used. We mainly used dorsal plates and structural bone grafts for conversion of arthroplasty to arthrodesis. The fusion rate in our converted arthrodesis group was moderately high with bone fusion established in 25 of 32 of the wrists (78%). These results are in accordance with those of other studies, indicating that the combination of rigid stabilization with a dorsal locking plate and structural bone grafting is necessary to achieve high fusion rates.

When compared with the literature, the fusion rate (34 of 39 wrists (87%)) in our primary arthrodesis group was lower than the union rate of 98% reported in a systematic review, which included 669 cases (Berber et al., 2018), 100% union in 39 cases reported by Meads et al. (2003) and 97% in 115 cases reported by Rauhaniemi et al. (2005). We defined bone fusion as partial bridging of trabecular bone at both radiocarpal and midcarpal levels. This criterium for bone union is not mentioned nor maintained in most articles. For example, Rauhaniemi et al. (2005) reported the union rate of 97% despite the fact that 35% of the midcarpal and third carpometacarpal joints did not fuse.

Reigstad et al. (2017) found a Patient-Rated Wrist and Hand Evaluation (PRWHE) score of 29 (SD 20) after 6.4 years of follow-up in his series. This differs somewhat from our findings, where patients with conversion to arthrodesis had a PRWHE-score of 43 (SD 25) and patients with primary arthrodesis had a PRWHE-score of 38 (SD 18). This difference in outcome could be attributed to the heterogeneity between the studies. When examining the baseline characteristics, our study population included more rheumatoid patients. Generally speaking, these patients suffer from a more widespread involvement of joints, resulting in an increased disability in daily activities. The high satisfaction rate in our study after a mean follow-up of 5 years is in line with that of Carlsson and Simmons (1998), who also reported a high satisfaction rate.

Conversion of a failed TWA to arthrodesis is not without risk of complications. Infection, hardware problems or carpal tunnel syndrome are serious complications that have been previously described (Beer and Turner, 1997; Carlson and Simmons, 1998). In our series, similar complications occurred in the converted arthrodesis group: tendon irritation or rupture being the most common, followed by nonunion. One wrist had apparently achieved radiographic fusion, but after plate removal nonunion became evident. Therefore, we suggest the more specific computed tomography to determine fusion before plate removal.

There are several limitations to this study inherent to the retrospective design. First, preoperative data including patient-reported outcomes were not available. Second, our data was collected from two different hospitals with two different surgeons and surgical techniques. Lastly, several patients declined participation, possibly resulting in selection bias. Furthermore, there were significant differences in demographics between the two groups, regarding gender, rheumatoid arthritis as underlying disease and the use of disease-modifying anti-rheumatic drugs (DMARDs). However, the groups were too small to perform a subanalysis for these variables. Further research with a larger sample size is required to determine if there is a difference in radiographic and clinical outcomes between rheumatoid and non-rheumatoid patients, and between bone grafting techniques. Finally, another drawback of our study is the size of our dataset, which was too small to prove a statistical difference in our outcome measures. Adequately powered, prospective studies are necessary for the best clinical translation of the outcomes. Regardless of some of these limitations, this remains a comparative study with a relatively large sample size.

In this study, despite the relatively high complication rates found in both groups, the results were slightly in favour of patients with a primary wrist arthrodesis. Therefore, we conclude that the timing, primary or conversion, of total wrist arthrodesis could influence patient outcomes. Patients should be aware of the high risk of complications after wrist arthrodesis.
